# Supplementary material for: The Effect of Silencing Fatty Acid Elongase 4 and 6 Genes on the Proliferation and Migration of Colorectal Cancer Cells
Source: Int J Mol Sci. 2023 Dec 18;24(24):17615. doi: 10.3390/ijms242417615 (PMC10743756; doi:10.3390/ijms242417615)
Supplement: Supplementary file 1 [file ijms-24-17615-s001.zip › ijms-2766788-supplementary.pdf]

International Journal of *Molecular Sciences*

MDPI

Communication

The Effect of Silencing Fatty Acid Elongase 4 and 6 Genes on the Proliferation and Migration of Colorectal Cancer Cells

Aleksandra Czumaj [1,*], Jarosław Kobiela [2,3], Adriana Mika [1], Emmanouil Pappou [4] and Tomasz Śledziński [1]

[1] Department of Pharmaceutical Biochemistry, Faculty of Pharmacy, Medical University of Gdansk, 80-211 Gdansk, Poland; adriana.mika@gumed.edu.pl (A.M.); tomasz.sledzinski@gumed.edu.pl (T.Ś.)
[2] Department of General, Endocrine and Transplant Surgery, Faculty of Medicine, Medical University of Gdansk, 80-211 Gdansk, Poland; jaroslaw.kobiela@gumed.edu.pl
[3] Department of Surgical Oncology, Faculty of Medicine, Medical University of Gdansk, 80-211 Gdansk, Poland
[4] Department of Surgery, Memorial Sloan Kettering Cancer Center, New York, NY 10065, USA; pappoue@mskcc.org
* Correspondence: aczumaj@gumed.edu.pl

Citation: Czumaj, A.; Kobiela, J.; Mika, A.; Pappou, E.; Śledziński, T. The Effect of Silencing Fatty Acid Elongase 4 and 6 Genes on the Proliferation and Migration of Colorectal Cancer Cells. *Int. J. Mol. Sci.* **2023**, *24*, x. https://doi.org/10.3390/xxxxx

Academic Editor: Rafael Coveñas Rodríguez

Received: 26 November 2023
Revised: 14 December 2023
Accepted: 15 December 2023
Published: 18 December 2023

Copyright: © 2023 by the authors. Submitted for possible open access publication under the terms and conditions of the Creative Commons Attribution (CC BY) license (https://creativecommons.org/licenses/by/4.0/).

Table S1. Relative expression level of selected fatty acid elongase in control conditions (basic culture medium), in the presence of positive siRNA control (siRNA GAPDH), negative siRNA control (siRNA(-)) and lipofectamine. Data presented as mean ± SD. * p<0.05 compared to control condition (base medium).

	ELOVL1	ELOVL2	ELOVL3	ELOVL4	ELOVL5	ELOVL6
			**CCD-841-CoN**			
Control	1.01±0.09	1.03±0.15	1.13±0.05	1.02±0.12	1.05±0.19	1.02±0.11
siRNA(-)	1.00±0.04	1.15±0.18	1.06±0.15	1.22±0.13	0.99±0.11	0.92±0.05
siRNA GAPDH	0.97±0.13	1.00±0.19	1.05±0.05	1.08±0.14	1.08±0.07	1.10±0.11
Lipofectamine	1.04±0.09	1.07±0.17	1.06±0.13	1.03±0.05	0.99±0.10	0.97±0.08
			**HT-29**			
Control	1.01±0.10	1.02±0.06	1.06±0.16	1.11±0.21	1.14±0.17	1.04±0.15
siRNA(-)	1.04±0.27	1.08±0.05	1.23±0.03	0.94±0.05	1.05±0.18	1.03±0.17
siRNA GAPDH	1.13±0.05	1.18±0.11	1.19±0.07	1.09±0.15	0.95±0.10	1.15±0.24
Lipofectamine	1.16±0.22	0.96±0.08	1.04±0.09	1.25±0.11	1.01±0.15	0.97±0.11
			**WiDr**			
Control	1.01±0.04	1.02±0.03	1.01±0.02	1.01±0.08	1.00±0.04	1.00±0.10
siRNA(-)	1.12±0.12	0.99±0.22	0.95±0.11	1.10±0.12	0.98±0.06	1.16±0.06
siRNA GAPDH	1.13±0.03	1.14±0.06	1.06±0.07	1.13±0.06	1.15±0.12	1.11±0.06
Lipofectamine	0.99±0.01	1.12±0.17	1.20±0.14	1.17±0.11	1.04±0.14	0.98±0.03

Table S2. Cell viability (%) after 72h incubations with lipofectamine, negative control, and positive control. Data presented as mean ± SD. * p<0.05 compared to control condition (base medium).

Culture conditions	Cell line type		
	CCD-841-CoN	HT-29	WiDr
Control	100±4.58	100±8.87	100±8.25
Lipofectamine	98.69±7.45	95.05±8.86	99.15±4.45
siRNA(-)	98.46±7.84	92.14±1.31	99.75±2.95
siRNA GAPDH	42.20±7.82 *	27.25±7.11 *	23.54±3.64 *

Table S3. The migration rate [%] of CCD-841-CoN, HT-29, and WiDr at 0h, 24h, 48h and 72h incubation with negative control (siRNA(-)) or lipofectamine. * p<0.05 compared to control condition (base medium).

	0h	24h	48h	72h
	**CCD-841-CoN**			
Control	0.00	40.00±2.95	55.5±1.52	100.00±4.36
siRNA(-)	0.00	32.8±4.37	63.08±2.73	97.62±2.55
Lipofectamine	0.00	36.7±4.74	66.9±4.93	99.62±1.52
	**HT-29**			
Control	0.00	40.02±2.36	63.00±4.76	100.00±3.65
siRNA(-)	0.00	44.3±7.81	66.1±2.91	97.4±5.58
Lipofectamine	0.00	46.84±2.45	62.62±4.84	96.39±4.32
	**WiDr**			
Control	0.00	33.8±1.69	75.6±4.42	100.00±3.66
siRNA(-)	0.00	35.6±2.26	76.3±3.84	97.26±4.51
Lipofectamine	0.00	30.40±2.25	75.50±2.51	92.36±5.58
